# The m^6^A methyltransferase METTL3 in intervertebral disc degeneration: from molecular mechanisms to therapeutic potential

**DOI:** 10.3389/fcell.2026.1949071

**Published:** 2026-09-30

**Authors:** Yi-Chun Zhou, Xiang-Song Xiang, Geng Li, Jian Zhang, Jun Ou

**Affiliations:** 1 Department of Spine Surgery, The Affiliated Nanhua Hospital, Hengyang Medical School, University of South China, Hengyang, Hunan, China; 2 Department of Orthopaedics, Danzhou People’s Hospital, Danzhou, Hainan, China; 3 Department of Spine Surgery, The First People’s Hospital of Chenzhou, Chenzhou, Hunan, China; 4 Orthopedic Center, The First Affiliated Hospital, Hengyang Medical School, University of South China, Hengyang, Hunan, China

**Keywords:** apoptosis, cell senescence, extracellular matrix, IDD, m^6^A, METTL3

## Abstract

Intervertebral disc degeneration (IDD) is the fundamental pathological process underlying low back pain, a major cause leading to disability worldwide. As the most prevalent methylation modification in eukaryotic RNA molecules, N6-methyladenosine (m^6^A) is involved in various diseases and cellular processes by regulating RNA fate. Methyltransferase-like protein 3 (METTL3) is the sole catalytic component of the methyltransferase complex and mediates m^6^A deposition on target RNAs. METTL3 is ubiquitously expressed and modulated by a variety of factors at the transcriptional and post-transcriptional levels. METTL3 expression is significantly increased in degenerative nucleus pulposus tissue and shows a positive correlation with degeneration degree. Importantly, recent studies have demonstrated that METTL3-mediated m^6^A modification is able to promote the progression of IDD through multiple approaches, including extracellular matrix metabolism imbalance, inflammatory response, nucleus pulposus cell senescence and apoptosis, and mitochondrial dysfunction. Thus, METTL3 could represent a promising therapeutic target for IDD. This review systematically summarizes the latest advances regarding the role of METTL3 in IDD and underlying molecular mechanisms, and also highlights its potential translational implications.

## Graphical Abstract

**Figure:**
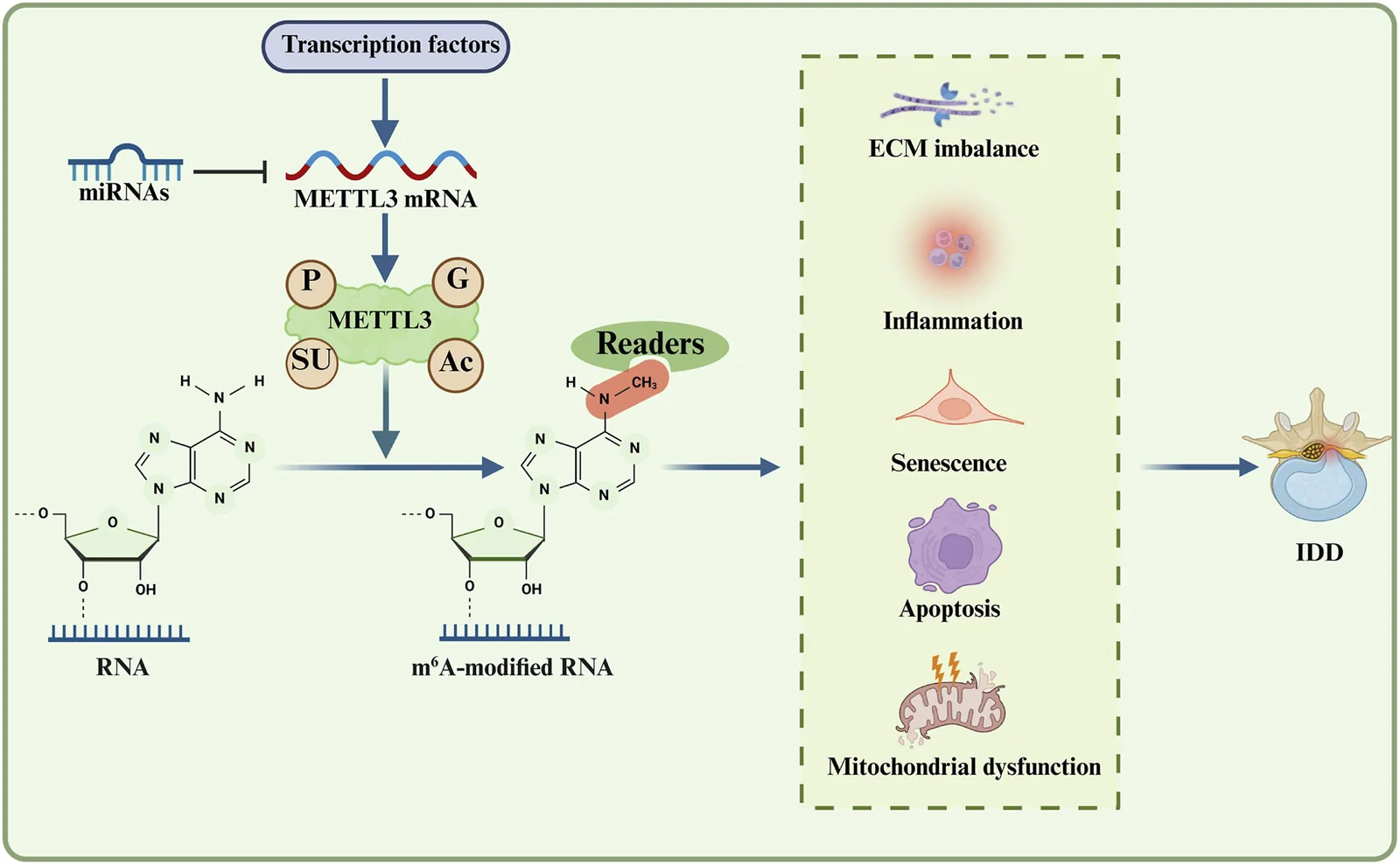

## 1 Introduction

Low back pain (LBP) is regarded as a leading cause of disability across the world and poses a heavy burden on the socioeconomic and medical systems. It is estimated that more than 80% of adults have to undergo LBP at least once during their lifetime, and about 23% of them will continue to suffer from the work-life challenges caused by LBP (Hoy et al., 2012). Intervertebral disc (IVD) lies between two adjacent vertebral bodies and is composed of three morphologically distinct regions: the central nucleus pulposus (NP), surrounding annulus fibrosus (AF), and two cartilaginous endplates (CEPs). IVD is essential to cushion loads and promote body movements. Nevertheless, IVD is prone to suffer from degeneration under stimulation of various harmful factors such as aging, trauma and excessive loading. IVD degeneration (IDD) constitutes the major pathological basis of LBP. As a complex pathological process, IDD is characterized by loss of IVD tissue structural integrity (Sloan et al., 2020). With the aggravation of aging and alterations of lifestyle, the incidence of IDD continues to increase in recent years. Currently, the therapeutic methods for IDD in clinic are still limited to drug treatments or surgical interventions aiming to improve clinical symptoms rather than delay or reverse the degenerative process (Xin et al., 2022). Several biological strategies, including injection of growth factors, gene therapy, stem cells, and exosomes, have shown considerable promise for slowing down IDD or stimulating IVD regeneration (Banala et al., 2019; Chen et al., 2025a; Fu et al., 2025; Muresanu et al., 2021). However, their long-term efficacy and safety still need to be carefully evaluated in clinical settings. Thus, it is imperative to strengthen the understanding about the pathophysiology of IDD, thereby helping to identify potential biomarkers for diagnosis and develop novel therapeutic targets for interventions.

Epigenetics refers to heritable alterations in gene expression without changing the DNA sequence, including DNA methylation, histone modifications, RNA modifications and chromatin remodeling. Increasing evidence has established that RNA epigenetic regulation plays a key role in the pathogenesis of musculoskeletal degeneration (Li et al., 2022c; Han et al., 2022). With the development high-throughput sequencing technologies, over 170 RNA epigenetic modifications have been identified, with N6-methyladenosine (m^6^A) being the most prevalent type in eukaryotic cells (Delaunay and Frye, 2019). The m^6^A modification is defined as RNA methylation occurring on the sixth nitrogen atom of adenine and plays a crucial role in a wide range of physiological and pathological processes, such as embryonic stem cell differentiation, meiosis, inflammatory response, oxidative stress and tumorigenesis (Wang et al., 2026; Xiong et al., 2025; Zhu et al., 2024; Chen et al., 2026; Yang et al., 2025a). Methyltransferase-like protein 3 (METTL3) functions as the sole catalytic component of the methyltransferase complex and mediates the deposition of m^6^A on the RRACH motif. There is increasing evidence that dysregulation of METTL3-mediated m^6^A modification is involved in the occurrence and development of a variety of diseases, including IDD (Yin et al., 2026; Liu et al., 2025a).

To comprehensively and systematically evaluate the relationship of METTL3 with IDD, we used the search terms “METTL3, m^6^A and intervertebral disc degeneration” in the PubMed database. We looked up 16 publications ranging from 2021 to 2026. After reading the abstract, we first ruled out the irrelevant articles. Subsequently, we thoroughly reviewed the remaining papers and categorized them according to the molecular mechanisms involved in IDD. Overall, this review aims to offer a useful framework for understanding the emerging role of METTL3 in IDD and its potential translational implications.

## 2 Overview of m^6^A modification

The m^6^A methylation predominantly distributes in 5′-untranslated region (UTR), the coding sequence and 3′-UTR of mRNAs, but also exists other types of RNA molecules, such as transfer RNAs (tRNAs), ribosomal RNAs (rRNAs), microRNAs (miRNAs), circular RNAs (circRNAs) and long noncoding RNAs (lncRNAs). The presence of m^6^A on the transcripts exerts a significant effect on RNA fate, including RNA splicing, processing, export, stabilization and translation (Jiang et al., 2021). As a dynamic and reversible process, m^6^A is installed by methyltransferases (writers), removed by demethylases (erasers) and recognized by binding proteins (readers). These m^6^A regulators and their biological functions are schematically presented in Figure 1.

**FIGURE 1:**
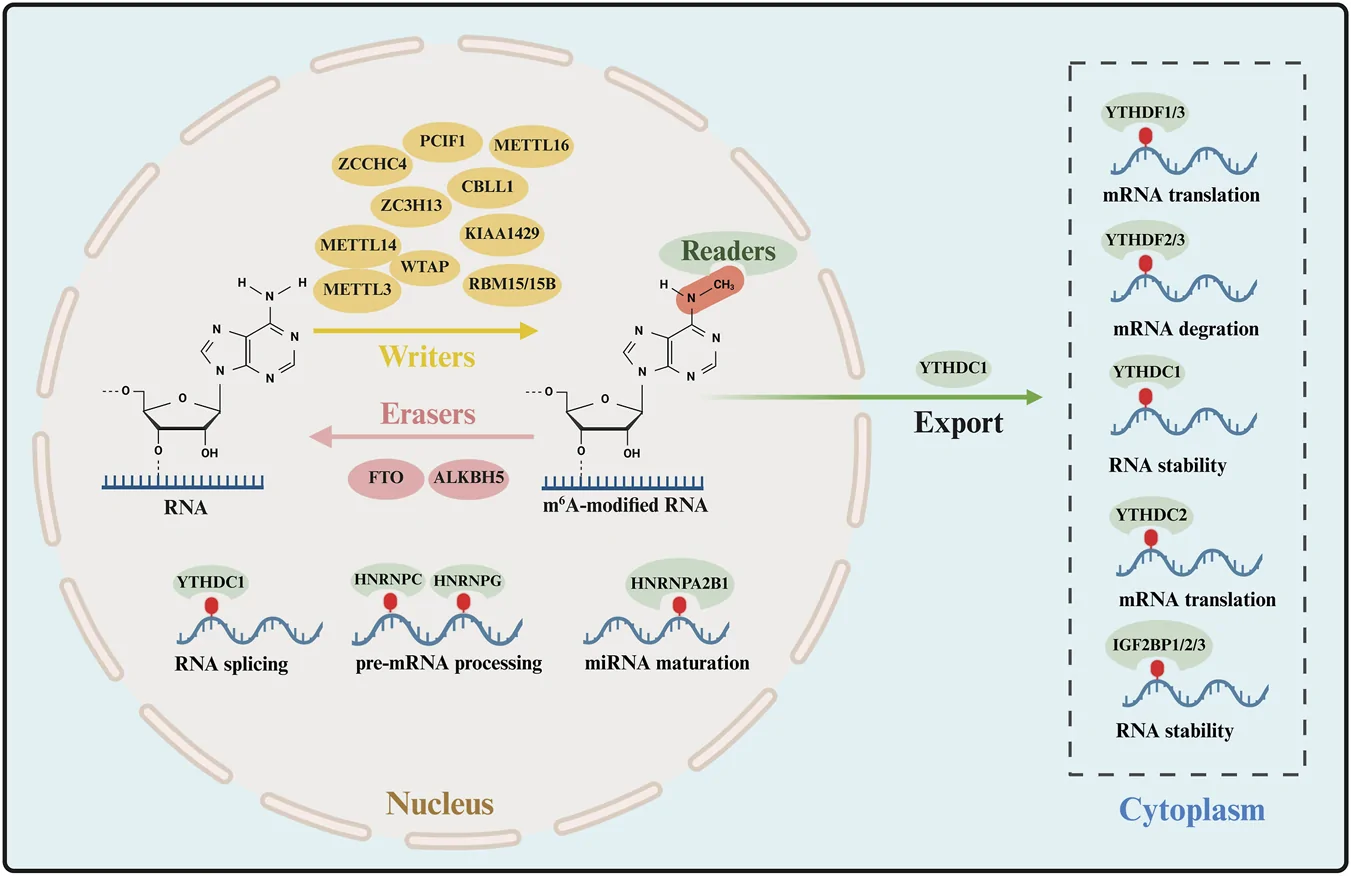
The molecular mechanism and biological functions of m^6^A modification. The m^6^A modification refers to the methylation of nitrogen at the sixth position of adenosine within RNA molecules. It is installed by methyltransferases (writers), removed by demethylases (erasers) and recognized by binding proteins (readers). The writers predominantly encompass METTL3, METTL14, WTAP, KIAA1429, RBM15/5B, ZC3H13, ZCCHC4, PCIF1, CBLL1 and METTL16. The erasers comprise FTO and ALKBH5. After recognition and binding by YTHs, HNRNPs or IGF2BPs, m^6^A exerts a significant effect on various aspects of RNA metabolism, including splicing, transport, stability and translation.

### 2.1 Writers and erasers

The m^6^A writers mediate the transfer of a methyl group from S-adenosylmethionine (SAM) to the N6 position of adenosine residues in RNA substrates for m^6^A formation. In mammals, the most well-investigated m^6^A writer is the methyltransferase-like protein 3 (METTL3)/METTL14/Wilms tumor 1-associated protein (WTAP) complex. METTL3 functions as a catalytic subunit, and METTL14 can enhance substrate recognition and RNA binding (Liu et al., 2014). WTAP is implicated in the recruitment of the METTL3/METTL14 heterodimer to target RNAs (Luo et al., 2026). The other known m^6^A methyltransferases encompass Vir-like m^6^A methyltransferase associated protein (VIRMA)/KIAA1429, RNA-binding protein 15/15B (RBM15/15B), zinc finger CCCH domain-containing protein 13 (ZC3H13), zinc finger CCHC-type containing 4 (ZCCHC4), phosphorylated C-terminal domain-interacting factor 1 (PCIF1), Cbl proto-oncogene like 1 (CBLL1) and METTL16 (Ping et al., 2014; Zhang et al., 2026c). Contrary to the effect of methyltransferases, demethylases can eliminate the m^6^A group from m^6^A-modified RNA molecules to render the process reversible. Currently, both fat mass and obesity-associated protein (FTO) and AlkB homolog 5 (ALKBH5) have been identified as the m^6^A erasers (Azzam et al., 2022). However, it remains unclear whether other enzymes are involved in m^6^A demethylation besides FTO and ALKBH5 (Azzam et al., 2022; Qu et al., 2022). Identification of new m^6^A demethylases will not only deepen our understanding of RNA epigenetic regulation but also help develop m^6^A-based therapies.

### 2.2 Readers

The m^6^A readers are a group of proteins that specifically recognize and bind directly to m^6^A-modified sites on RNA molecules, thereby functioning as the key regulators of the biological effects of m^6^A modification. The most well-characterized m^6^A readers are YT521-B homology (YTH) domain family proteins, including YTHDF1/2/3 and YTHDC1/2. YTHDF1 is known to enhance the translation efficiency of m^6^A-modified mRNAs, whereas YTHDF2 promotes the degradation of target mRNAs (Zaccara et al., 2019). YTHDF3 works in concert with YTHDF1 to facilitate protein translation; however, the cooperation of YTHDF3 with YTHDF2 contributes to mRNA decay (Shi et al., 2017). YTHDC1 contributes to RNA splicing, transport and stabilization, while YTHDC2 promotes the translation and protein expression of target mRNAs (Xiao et al., 2016; Wu et al., 2024c). The other known m^6^A readers include heterogeneous nuclear ribonucleoprotein family (HNRNPA2B1, HNRNPC, and HNRNPG) and insulin-like growth factor 2 mRNA-binding protein family (IGF2BP1/2/3). It has been demonstrated that HNRNPA2B1 can bind to m^6^A-bearing primary miRNAs for miRNA maturation by interacting with DiGeorge syndrome critical region gene 8 (DGCR8), the miRNA microprocessor complex protein (Alarcón et al., 2015). HNRNPC and HNRNPG bind directly to m^6^A-methylated pre-mRNAs, contributing to alternative splicing and target gene expression (Li et al., 2026; Zhou et al., 2019). In addition, IGF2BP1/2/3 are capable for enhancing the stability of m^6^A-modified RNAs (Duan et al., 2024).

## 3 Molecular structure and biochemical characteristics of METTL3

The genomic and protein information of METTL3 in different species, including *Homo sapiens, Sus scrofa, Mus musculus, Rattus norvegicus Xenopus laevis* and *Danio rerio*, is presented in Table 1. The human *METTL3* gene is located on chromosome 14q11.2, spans approximately 13.2kb, and encompasses 11 exons and 10 introns. The full-length METTL3 protein contains 580 amino acid residues with a molecular weight of 64 kDa. The mouse *METTL3* gene is mapped to chromosome 14qC1, spans about 10.3 kb, also consists of 11 exons and 10 introns, and encodes a 64 kDa protein containing 580 amino acid residues. However, the rat *METTL3* gene is located on chromosome 15p14, spans approximately 14.5 kb, comprises 12 exons and 11 introns. Likewise, METTL3 encoded-protein is made up of 580 amino acids, and its molecular weight is 64 kDa. Of note, the *METTL3* gene in various species has similar exon and intron structures, suggesting evolutionary preservation of gene organization. Full-length METTL3 protein sequence alignment showed that human METTL3 shares the highest sequence identity with *Sus scrofa* (98.28%), followed by *M. musculus* (97.07%), *R. norvegicus* (96.72%), *X. laevis* (77.68%), and *D. rerio* (70.90%), which is showed in Figure 2. This provides further evidence that METTL3 is highly conserved throughout evolution. Taken together, this high degree of conservation in gene and protein reveals that METTL3 may possess similar biological functions and regulatory mechanisms across species.

**TABLE 1:** Information of METTL3-encoding gene from different sources.

METTL3 source	Chromosomal location	Full length (bp)	Intron	Exon	Number of amino acids	Molecular weight (KDa)
*Homo sapiens*	14q11.2	13203	10	11	580	64
*Sus scrofa*	7	17515	10	11	580	64
*Mus musculus*	14qC2	10288	10	11	580	64
*Rattus norvegicus*	15p14	14476	11	12	580	64
*Xenopus laevis*	1L	21848	10	11	573	63
*Danio rerio*	7	10524	12	13	584	64

**FIGURE 2:**
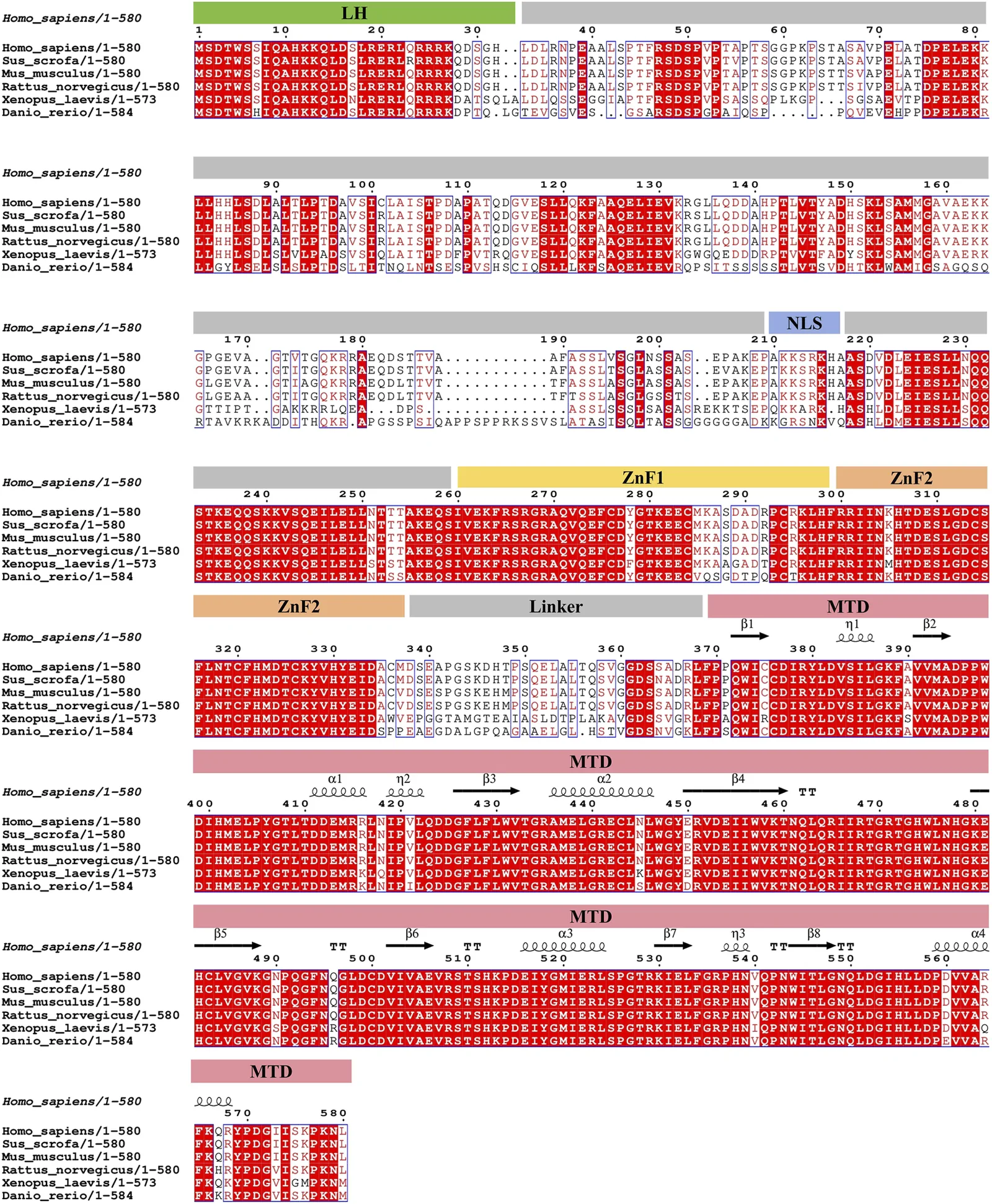
Multiple sequence alignment of METTL3 protein in various sources. The amino acid sequence of full-length METTL3 protein is derived from *Homo sapiens* (NP_062826.2), *Sus scrofa* (XP_003128628.1), *Mus musculus* (NP_062695.2), *Rattus norvegicus* (NP_001019965.1), *Xenopus laevis* (NP_001084701.1) and *Danio rerio* (NP_997945.1). Conserved amino acid residues are shaded in red background. The top of sequence represents the secondary structure of METTL3 from *Homo sapiens*. The β-sheets are shown as arrows, and strict β turns are designated as TT.

Structurally, human METTL3 protein predominantly comprises a leading helix (LH), a nuclear localization signal (NLS), a zinc finger domain (ZFD), a methyltransferase domain (MTD), and a linker connecting ZFD and MTD (Figure 3). Both LH and NLS work in concert with other members of the methyltransferase complex to promote the translocation of METTL3 from the cytoplasm into the nucleus. ZFD contains two consecutive CCCH-type zinc fingers, known as ZnF1 and ZnF2. These two zinc fingers are connected by an antiparallel β-sheet (Huang et al., 2019). ZFD plays a critical role in the specifically recognizing RNA substrates and stabilizing the catalytic activity of the methyltransferase complex (Huang et al., 2019). The linker serves as a bridge linking ZFD to MTD and is flexible but partially folded, contributing to the cooperation between the two domains during the process of catalysis.

**FIGURE 3:**
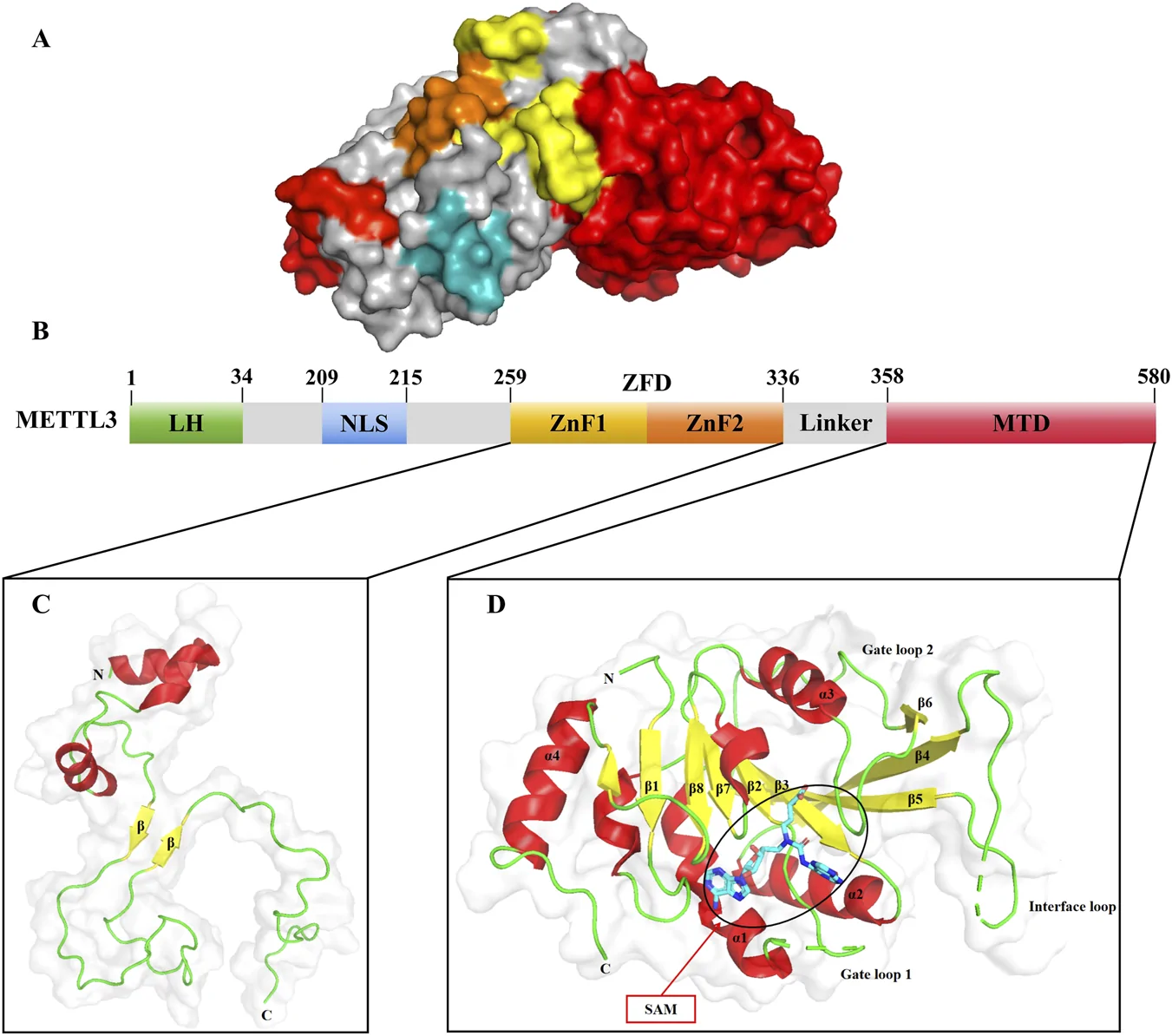
Structural features of METTL3. **(A)** The surface representation of METTL3 domains. **(B)** The domain architecture of MET1L3. **(C)** Schematic diagram of the three-dimensional structure of ZFD (PDB ID:5yz9). **(D)** Schematic presentation of the three-dimensional structure of MTD (PDB ID: 8PWB).

The MTD of METTL3, also known as MT-A70, serves as the catalytic core for m^6^A methylation. It adopts a canonical Rossmann fold and belongs to the Class I methyltransferase superfamily (Wang et al., 2016b). This domain is made up of four α helices (α1, α2, and α4 on one side, and α3 on the other), eight β folds, three 3_10_ helices and three loops (Gate loop 1, interface loop and Gate loop 2). These elements form a classical α-β-α sandwich fold (Corbeski et al., 2024; Ruszkowska et al., 2018). Gate loop 1 and gate loop 2 are adjacent to the SAM-binding pocket. Gate loop 1 contains a conserved DPPW motif (residues 395–399) as the methyltransferase active site (Yuan et al., 2024). After m^6^A installation, Gate loop 1 and Gate loop 2 undergo significant conformational changes, resulting in closure of the cofactor-binding pocket to avoid excessive methylation. Therefore, both loops play a critical role in regulating the catalytic activity (Wang et al., 2016b). The interface loop, which is located at the boundary between METTL3 and its interacting proteins, is indispensable for the formation of the METTL3/METTL14 heterodimer (Wang et al., 2016a). Collectively, this unique structural configuration of MTD distinguishes METTL3 from other members of the METTL family and may determine its specialized functional roles.

## 4 Expression and modulation of METTL3

METTL3 is broadly expressed in almost all cells, tissues and organs such as macrophages, adipose tissue, heart, brain and kidney, underscoring its biological importance. Furthermore, the vast majority of METTL3 is located in the nucleus and only a small amount of METTL3 exists in the cytoplasm (Lin et al., 2016). The expression of METTL3 is regulated by a variety of factors at the transcriptional and post-transcriptional levels (Figure 4).

**FIGURE 4:**
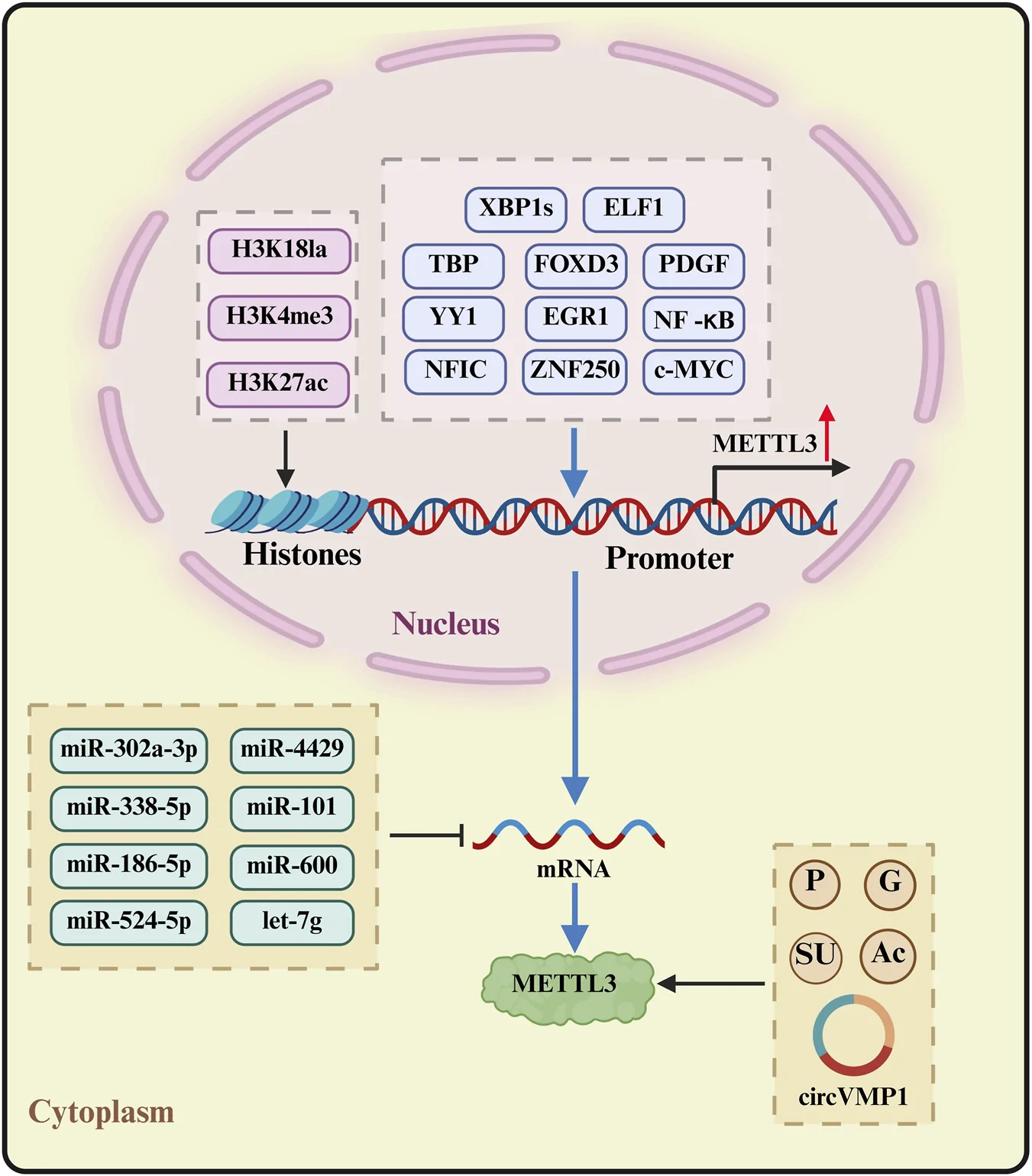
Schematic diagram of METTL3 regulators. METTL3 is transactivated by histone modifications (H3K18la, H3K4me3 and H3K27ac) and transcription factors (XBP1s, ELF1, TBP, FOXD3, PDGF, YY1, EGR1, NF-κB, NFIC, ZNF250 and C-MYC). MiRNAs (miR-302a-3p, miR-524-5p, miR-186-5p, miR-4429, let-7g, miR-600 and miR-101, miR-338-5p) downregulates METTL3 expression by binding to its 3′-UTR. METTL3 protein is stabilized by phosphorylation, O-GlcNAcylation, SUMOylation and acetylation. Also, circMVP interacts with METTL3 protein to enhance its stability. P: phosphorylation, G: O-GlcNAcylation, SU: SUMOylation, Ac: acetylation.

### 4.1 Transcription factors

In the METTL3 promoter region, there is a variety of potential binding sites of transcription factors. Consistently, multiple transcription factors involving METTL3 transcription have been identified. For instance, spliced X-box binding protein 1 (XBP1s), E74-like factor 1 (ELF1), TATA-binding protein (TBP), forkhead box D3 (FOXD3), platelet-derived growth factor (PDGF), Yin Yang 1 (YY1) and early growth response 1 (EGR1) can bind directly to the METTL3 promoter and consequently upregulate its expression (Wang et al., 2024a; Liu et al., 2025c; Li et al., 2020; Chen et al., 2022b; Lv et al., 2022; Li et al., 2022d; Dong et al., 2023). Steroid receptor coactivator-1 (SRC-1) interacts with the p50 subunit of nuclear factor-κB (NF-κB). The p65 subunit then binds to the METTL3 promoter for transcriptional activation (Liu et al., 2025b). Exposure to cigarette smoke condensate substantially decreases the methylation levels within the CpG island of the *METTL3* gene, which recruits transcription factor NFIC (nuclear factor I-C) to augment METTL3 expression in pancreatic ductal adenocarcinoma cells (Zhang et al., 2019). LncRNA AC116914.2 competitively binds to miR-1245a to upregulate the expression of zinc finger protein 250 (ZNF250), which facilitates METTL3 transcription (Feng et al., 2025). In addition, c-MYC transcriptionally activates METTL3, which enhances LINC01006 stability in an m^6^A-dependent manner and promotes non-small cell lung cancer progression (Liu et al., 2024a). It is worth noting that LINC01006 can also upregulate c-MYC expression by sequestering miR-34 and miR-2682 (Liu et al., 2024a). Thus, there is a positive feedback loop among c-MYC, METTL3 and LINC01006.

### 4.2 Histone modifications

Histones function as the fundamental building blocks of chromatin and comprise four components: H2A, H2B, H3 and H4. The N- and C-terminal tails of these histone components undergo various post-translational modifications, which can alter the spatial structure of chromatin and profoundly influence gene expression (Yao et al., 2024). Histone modifications, such as lactylation acetylation, methylation and acetylation, have been shown to exert a significant effect on METTL3 transcription.

Lactylation, a highly evolutionarily conserved process, refers to the addition of a lactyl group to lysine residues of histones or non-histone proteins using lactate as a substrate (Fan et al., 2023). It has been demonstrated that overexpression of lactate dehydrogenase A (LDHA), a critical regulator of the Warburg Effect by catalyzing the conversion of pyruvate to lactate in the final step of glycolysis, reinforces the enrichment of histone H3 lysine 18 lactylation (H3K18la) in the METTL3 promoter region to provoke its transcription in gastric cancer cells (Qian et al., 2025). Similar to this report, high lactate increases H3K18la levels in a P300-dependent mechanism to stimulate METTL3 transcription in alveolar epithelial cells (Wu et al., 2024a). Elevated H3K18la levels are observed in ossification of the ligamentum flavum (OLF) patients (Zhou et al., 2025). Knockdown of LDHA counteracts METTL3 transcription by inhibiting H3K18la accumulation in OLF cells (Zhou et al., 2025).

Like histone lactylation, histone methylation is also an important and widespread type of chromatin modification (Greer and Shi, 2012). In intrahepatic cholangiocarcinoma cells, SET domain containing 1B was found to mediate trimethylation of lysine 4 on histone 3 (H3K4me3) in the METTL3 promoter region, leading to its transcriptional activation (Xu et al., 2022). E−26 transformation specific-1 interacts with WD repeat domain 5, which then binds to the METTL3 promoter for H3K4me3 modification and stimulates its transcription in cervical cancer cells (Du et al., 2022). Notably, METTL3 is able to diminish the enrichment of H3K4me3 in embryonic stem cells (Liu et al., 2022b). This suggests that a negative feedback loop may exist between METTL3 and H3K4me3 to avoid excessive METTL3 upregulation.

Histone acetylation predominantly occurs at lysine residues of histones and plays a critical role in regulating gene activity (Kablan et al., 2025). In gastric cancer cells, P300-mediated acetylation of lysine 27 on histone 3 (H3K27ac) in the promoter of METTL3 was shown to activate its transcription (Wang et al., 2020). Curiously, overexpression of METTL3 can downregulate P300 expression in an m^6^A-dependent manner to inhibit H3K27ac modification in human pluripotent stem cells (An et al., 2025). These findings indicate that there is a cross-talk between H3K27ac and m^6^A.

### 4.3 Noncoding RNAs

Noncoding RNAs are a class of RNA molecules without protein-coding capacity and predominantly contain miRNAs, lncRNAs and circRNAs. These noncoding RNAs play an important role in regulating METTL3 expression at the post-transcriptional level. MiRNAs are short, noncoding, single-stranded RNAs that are composed of 20–25 nucleotides (Saliminejad et al., 2019). It has been reported that hepatitis B virus X-interacting protein (HBXIP) decreases miRNA let-7g abundance, leading to increased expression of its target gene METTL3 (Cai et al., 2018). MiR-302a-3p directly interacts with 3′-UTR of METTL3 to downregulate its expression, thereby inhibiting melanoma cell progression (Chang et al., 2022). Several other miRNAs, such as miR-186-5p, miR-4429, miR-600 and miR-101, have been confirmed to directly target METTL3 (Ma et al., 2024; He et al., 2019; Wei et al., 2019; Yan et al., 2025).

As a class of single-stranded RNA molecules, circRNAs are characterized by their covalently closed structures and are ubiquitous across species ranging from viruses to mammals (Zhou et al., 2020). CircMVP (hsa_circ_0000688) is generated from the eighth exon of major vault protein (MVP) pre-mRNA by backsplicing, with a spliced sequence length of 256nucleotides. It was found that circMVP interacts with METTL3 protein to inhibit ubiquitin proteasome system-mediated degradation in colorectal cancer cells, leading to increased METTL3 expression (Wang et al., 2025a). Competing endogenous RNAs (ceRNAs) are defined as RNA transcripts that cross-regulate each other by competing for shared miRNAs (Thomson and Dinger, 2016). CircRNA vacuole membrane protein 1 (circVMP1) acts as a ceRNA of miR-524-5p to facilitate METTL3 expression (Xie et al., 2022). LncRNAs are a class of RNA transcripts that are more than 200 nucleotides in length, and participate in the regulation of a variety of cellular processes (Kohlmaier et al., 2023). LINC00240, a recently discovered lncRNA, serves as a molecular sponge for miR-338-5p to upregulate METTL3 expression (Wang et al., 2021).

### 4.4 Post-translational modifications

The covalent binding of chemical groups or small molecule proteins to specific sites of amino acid sequences can result in post-translational modifications of proteins. This is thought to be a fundamental biological process that contributes to protein maturation and expression. Recent studies have demonstrated that METTL3 undergoes multiple post-translational modifications, including phosphorylation, O-GlcNAcylation, SUMOylation and acetylation.

Phosphorylation is an important biological event essential for regulating protein activity and stability, and plays a significance role in maintaining the physiological activities of cells. It was found that prevention of phosphatase 2A catalytic subunit Cα increases the activity of ataxia-telangiectasia mutated kinase, which mediates the phosphorylation of METTL3 at the S43 position and upregulates METTL3 expression (Cheng et al., 2021). Additionally, extracellular signal-regulated kinase 1/2 (ERK1/2) directly phosphorylates METTL3 at S43, S50 and S52. Its zinc finger domain then interacts with the deubiquitinase USP5 (ubiquitin specific peptidase 5) to reduce the ubiquitination and subsequent degradation of METTL3 (Sun et al., 2020). Of note, complement C5a receptor 1 (C5aR1), a typical G protein-coupled receptor, is able to promote the phosphorylation of ERK1/2 to enhance the METTL3 expression in glioblastoma cells (Meng et al., 2024). This provides another important mechanism by which ERK1/2 regulates METTL3 expression.

O-GlcNAcylation is involved in the enzymatic transfer of O-linked β-N-acetylglucosamine (O-GlcNAc) to the hydroxyl groups of serine and threonine residues in proteins (Hart et al., 2007). O-GlcNAc transferase (OGT)-mediated O-GlcNAcylation of METTL3 at Thr186, Ser192 and Ser193 sites dramatically suppresses its ubiquitination, thereby enhancing protein stability (Chen et al., 2025b). After O-GlcNAcylation at the Ser118 site, METTL3 interacts with eukaryotic translation initiation factor 3 subunit H (a deubiquitinase) to reduce its degradation mediated by ubiquitination (Wang et al., 2025b). Also, METTL3 is O-GlcNAcylated by OGT at Ser184 and Ser243. This O-GlcNAcylation is able to stabilize METTL3 protein by recruiting USP5, without effect on its interaction with METTL14 or WTAP (Gou et al., 2025).

SUMOylation is a reversible biological process involved in the covalent binding of small ubiquitin-like modifiers (SUMOs) to target proteins (Huang et al., 2024). It has been reported that SUMO1 mediates the SUMOylation of METTL3 at its lysine residues (K177, K211, K212 and K215) in non-small cell lung carcinoma cells, and this modification has no impact on its stability, subcellular localization and interaction with METTL14/WTAP but decreases the methyltransferase activity (Du et al., 2018). Stimulation with mitogen increases the expression of UBC9, which promotes the conjugation of SUMO1 with METTL3, causes the SUMOylation at K177, K211, K212 and K215 and consequently diminishes intracellular m^6^A abundance in hepatocellular carcinoma cells (Xu et al., 2020). However, another study showed that METTL3 is SUMOylated by SUMO1 at the K459 position in colorectal cancer cells, which promotes METTL3 accumulation and enhances circ_0000677 stability in a m^6^A-dependent manner (Liu et al., 2022a). These conflicting findings suggest that the effect of SUMOylation on METTL3 expression is complex, which is likely due to the differences in SUMOylation sites, research models and microenvironments. Future studies are required to determine the precise role of SUMOylation in regulating METTL3 expression.

In addition to histones, non-histones are frequently acetylated and constitute a primary portion of the acetylome in mammalian cells (Narita et al., 2019). Acetyl-CoA acetyltransferase 1 (ACAT1) acetylates METTL3 at K263 and K388 sites and thus stabilizes METTL3 protein by blocking the degradation of ubiquitin-proteasome (Zhang et al., 2023). Treatment with sulfatide inhibits histone deacetylase 2 expression and facilitates METTL3 acetylation, which reduces its binding to METTL14 and WTAP (Yang et al., 2022c). Interestingly, interleukin (IL)-6 promotes METTL3 deacetylation and nuclear translocation by upregulating the deacetylase sirtuin 1 expression, resulting in a marked increase in global m^6^A levels (Li et al., 2022e). Therefore, acetylation has a multifaceted effect on METTL3, including gene transcription, protein stabilization, subcellular distribution and interaction with other methyltransferase complex components.

## 5 Role of METTL3 in IDD

The cause of IDD is multifactorial and involves a complex interplay of environmental exposure, genetic predisposition and lifestyle factors (Hammoor et al., 2026). Recent studies have demonstrated that METTL3-mediated m^6^A modification exerts a significant effect on IDD progression through multiple mechanisms, including the imbalance of extracellular matrix (ECM) metabolism, inflammatory response, NP cell senescence and apoptosis, as well as mitochondrial dysfunction (Figure 5; Table 2).

**FIGURE 5:**
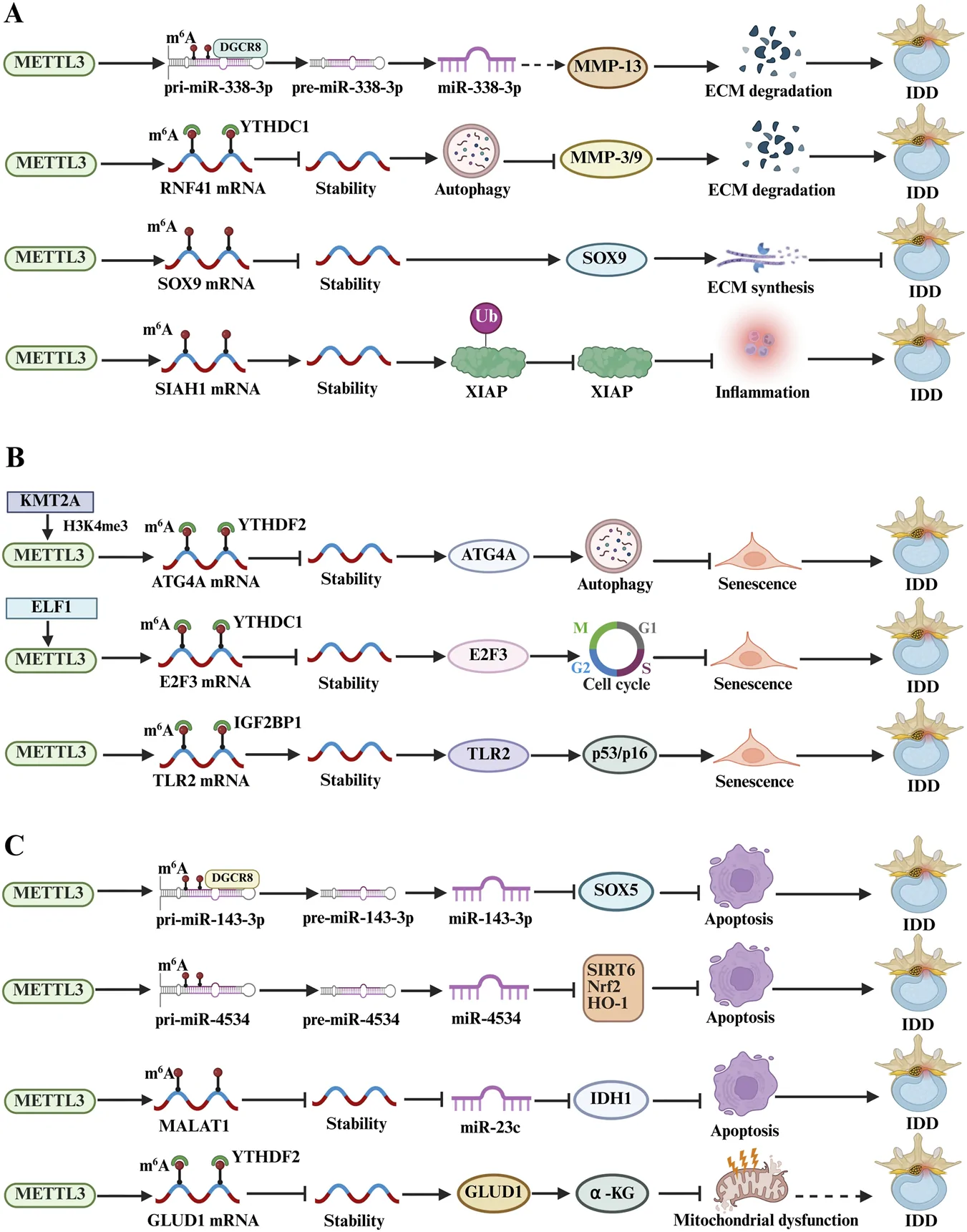
The effects and mechanisms of METTL3 on IDD. METTL3-mediated m^6^A modification promotes the development of IDD through multiple signaling pathways. **(A)** METTL3 facilitates ECM degradation by mediating the m^6^A modification of pri-miR-338-3p and RNF41, and suppresses ECM synthesis by destabilizing SOX9. METTL3 aggravates inflammation through the SIAH1/XIAP pathway. **(B)** METTL3 stimulates NP cell senescence by increasing the m^6^A levels of ATG4A, E2F3 and TLR2 transcripts. **(C)** METTL3 induces NP cell apoptosis by facilitating miR-143-3p and miR-4534 maturation as well as inhibiting MALAT1 production. In addition, METTL3 downregulates GLUD1 expression to promote mitochondrial dysfunction.

**TABLE 2:** The role of METTL3 in the pathogenesis of IDD.

Experimental models	Animals	Targets	Molecular mechanisms	Biological functions	References
Human NP cells	Rat	Pri-miR-338-3p	MiR-338-3p maturation by DGCR8	Promoting Col II degradation	Sloan et al. (2020)
Human NP cells	Rat	RNF41	RNF41 mRNA stabilization by YTHDC1	Promoting Col II and aggrecan degradation and inflammation	Sun et al. (2025)
Human endplate chondrocytes	Rat	SOX9	SOX9 mRNA destabilization	Blocking Col II synthesis	Wang et al. (2017)
Human NP cells	Not involved	SIAH1	SIAH1 mRNA stabilization	Accelerating inflammatory response	Wang et al. (2018)
Human NP cells	Rat	ATG4A	ATG4A mRNA destabilization by YTHDF2	Stimulating cell senescence	Wu et al. (2023)
Human NP cells	Rat	E2F3	E2F3 mRNA destabilization YTHDC1	Stimulating cell senescence	He et al. (2019)
Human NP cells	Rat	TLR2	TLR2 mRNA stabilization by IGF2BP1	Stimulating cell senescence	Wu et al. (2024c)
Human NP cells	Rat	Pri-miR-143-3p	MiR-143-3p maturation by DGCR8	Inducing cell apoptosis	Xie et al. (2022)
Human NP cells	Rat	Pri-miR-4534	MiR-4534 maturation	Inducing cell apoptosis	Xin et al. (2022)
Human NP cells	Mice	MALAT1	MALAT1 destabilization	Promoting cell apoptosis	Xu et al. (2022)
Human NP cells	Not involved	GLUD1	GLUD1 mRNA destabilization byYTHDF2	Promoting mitochondrial dysfunction	Yang et al. (2025b)

### 5.1 METTL3 promotes the imbalance between ECM anabolism and catabolism

The major ECM components within the IVD are collagens and proteoglycans, especially type II collagen (Col II) and aggrecan. In a healthy IVD, the anabolism and catabolism of ECM are in a dynamic balance, which is essential to maintain normal structure and functions of IVD. During the process of IDD, this homeostatic balance is disrupted by various stimuli and ultimately leads to decreased water absorption, a dysfunctional mechanical microenvironment and decreased resistance to the loading (Liang et al., 2022). Studies from our group and other teams have demonstrated that promoting ECM degradation aggravates IDD, whereas stimulation of ECM synthesis has an opposite effect (Wang et al., 2017; Wang et al., 2018; Liu et al., 2025d; Jin et al., 2024). Thus, progressive loss of ECM components is considered to be a critical pathological feature of IDD, and restoring the dynamic equilibrium of ECM metabolism is great importance for therapeutic intervention.

In recent years, the role of m^6^A in regulating ECM metabolism has gained considerable attention. NP cells are the major cell type resident in the IVD and play a critical role in the generation of ECM and maintenance of disc microenvironment (Silagi et al., 2018). A study from our laboratory showed that the expression of m^6^A regulators, such as METTL3, METTL14, WTAP, KIAA1429 and YTHDC1, is significantly increased in degenerated NP tissue of mice (Zhu et al., 2021). Higher levels of METTL3 is also observed in human degenerated NP tissue (Yang et al., 2025b). Matrix metalloproteinases (MMPs) and a disintegrin and metalloprotease with thrombospondin motifs (ADAMTSs) are known to be responsible for ECM degradation (Wang et al., 2015). Overexpression of METTL3 mediates m^6^A methylation of pri-miR-338-3p and promotes mature miR-338-3p production by interacting with DGCR8 in NP cells challenged with IL-1β. Subsequently, miR-338-3p increases MMP-13 expression, facilitates Col II degradation and eventually accelerates the progression of IDD in rats (Yang et al., 2025b). It is well known that miRNAs interact with 3′-UTR of target mRNAs to exert their biological effects (Vishnoi and Rani, 2023). Given that MMP-13 is not a direct target of miR-338-3p, future research needs to identify the target genes of miR-338-3p so as to elucidate how miR-338-3p regulates MMP-13 expression. Another study revealed that m^6^A modification of ring finger protein 41 (RNF41) transcripts induced by METTL3/YTHDC1 dramatically shortens its half-life, which disrupts autophagy, upregulates MMP-3 and MMP-9 expression, and diminishes Col II and aggrecan contents in degenerative NP cells (Yao et al., 2025). Moreover, intradiscal injection of adeno-associated virus encoding short hairpin RNA shRNA targeting METTL3 attenuates puncture-induced disc loss and histopathological degeneration in a rat model of IDD by, at least in part, restoring ECM metabolism balance (Yao et al., 2025). These findings obtained from cultured cells, animal models and human tissue suggest that targeting METTL3 could represent a promising new strategy for blocking ECM breakdown and treating IDD. Of note, METTL3 cooperates with IGF2BP2 to stabilize signal transducer and activator of transcription 1, thereby transactivating ADAMTS-12 in IL-1β-treated rat chondrocytes and promoting osteoarthritis progression (Yang et al., 2023). However, whether METTL3 promotes ECM catabolism and IDD development by regulating the expression of ADAMTSs remains unclear and needs to be investigated.

Sex-determining region Y-box transcription factor 9 (SOX9) is known to stimulate the transcription of Col II and aggrecan (Bell et al., 1997; Cheng et al., 2009). With the progression of IDD, the expression of SOX9 in human degenerated NP tissue shows a decreasing trend (Gruber et al., 2005). Deletion of SOX9 causes severe IDD through ECM remodeling in mice (Tsingas et al., 2020; Aboushaala et al., 2025). Interestingly, METTL3 expression and global m^6^A methylation levels are higher in human degenerative endplate cartilage tissue than those in controls (Xiao et al., 2022). Further studies revealed that METTL3 catalyzes the m^6^A modification of SOX9 mRNA, which in turn reduces its stability, inhibits Col II synthesis, and promotes IDD development in rats (Xiao et al., 2022). Thus, in addition to promoting ECM degradation, prevention of ECM biogenesis is another important mechanism by which METTL3-mediated m^6^A modification exacerbates IDD.

### 5.2 METTL3 amplifies inflammatory response

As a fundamental component of the host defense system, inflammation orchestrates immune cell recruitment, cytokine generation and tissue repair/regeneration in response to infection or injury. However, persistent or dysregulated inflammatory response contributes to the occurrence and development of many diseases, including IDD. Increased expression of pro-inflammatory cytokines, such as tumor necrosis factor-α (TNF-α), IL-1β, IL-6, IL-17 and IL-23, is frequently observed in the degenerative IVD (Shamji et al., 2010; Sadowska et al., 2018; Jiang et al., 2016). There is considerable evidence demonstrating that inflammatory response not only promotes ECM degradation, but also induces IVD cell senescence and death (Kang et al., 2023; Molinos et al., 2015). Recent studies have highlighted that METTL3 exerts a potent pro-inflammatory effect across multiple pathological contexts, predominantly by suppressing pro-inflammatory mediator production and regulating critical immune signaling pathways (Yang et al., 2022a; Wang et al., 2022a; Yin et al., 2024). Notably, METTL3-mediated inflammation is also associated with IDD. For instance, METTL3 methylates and stabilizes seven-absent homolog 1 (SIAH1) mRNA, which ubiquitinates X-linked inhibitor of apoptosis protein (XIAP) for degradation, promotes IL-1β and IL-6 production, and eventually accelerates IDD progression (Fang et al., 2022). In addition, METTL3-catalyzed m^6^A modification of RNF41 transcripts stimulates IL-1β and IL-18 release from NP cells to drive IDD (Yao et al., 2025). Taken together, these observations derived from *in vitro* experiments and *in vivo* validation have established METTL3 as a key modulator of inflammatory response in the context of IDD. Intervention strategy aiming to inhibit critical METTL3 signaling pathways, such as METTL3/SIAH1 and METTL3/RNF41, may help maintain immune homeostasis and prevent excessive inflammatory injury within the discs.

### 5.3 METTL3 stimulates NP cell senescence

Cellular senescence is generally regarded as a state of permanent cell cycle arrest that is accompanied by halted proliferation triggered by endogenous or exogenous stimuli. As an age-related chronic disease, IDD is closely associate with the senescence of IVD cells, especially NP cells. During the progression of aging, NP cells gradually become senescence under stimulation of environmental stress and intrinsic factors (Feng et al., 2016). The senescence of NP cells not only decreases the number of functional cells within the disc and promotes aberrant secretion of pro-inflammatory cytokines and MMPs, but also accelerates the senescent process of neighboring healthy cells (Silwal et al., 2023; Liang et al., 2026b). Prevention of NP cell senescence has been shown to mitigate IDD in animal models (Jia et al., 2024; Wang et al., 2024b). Thus, protection of NP cells against senescence has been proposed as a novel and promising strategy for IDD therapy.

The m^6^A modification plays an important role in regulating NP cell senescence. For instance, WTAP works in concert with YTHDF2 to destabilize lncRNA NORAD in an m^6^A-dependent manner, thereby promoting NP cell senescence and IDD development (Li et al., 2022b). ALKBH5 causes NP cell senescence and aggravates IDD by demethylating DNA methyltransferase 3B (DNMT3B) transcripts and in turn upregulating DNMT3B expression via less YTHDF2 recognition (Li et al., 2022a). Similarly, METTL3 expression and global m^6^A level gradually increase in rat NP tissue with age (Liu et al., 2024b). Further analyses revealed that during the progression of NP aging, there are 1126 persistently differentially m^6^A peaks within 931 genes, with 51 genes being persistently differential expression (Liu et al., 2024b). H3K4me3 is one of the most studied histone modifications due to its enrichment at the start sites of transcription and plays an important role in regulating gene expression (Wang and Helin, 2025). A recent study showed that lysine methyltransferase 2A (KMT2A) upregulates METTL3 expression through the H3K4me3 modification (Wu et al., 2024b). METTL3 then cooperates with YTHDF2 to destabilize autophagy-associated gene 4A (ATG4A) in an m^6^A-dependent manner, which impairs autophagy and promotes NP cell senescence and IDD development (Wu et al., 2024b). ELF1 is a transcription factor that belongs to the erythroblast transformation specific family. It was found that ELF1 can bind to the METTL3 and YTHDF2 promoter regions for transcriptional activation (Liu et al., 2025c). Subsequently, METTL3 mediates m^6^A methylation of E2F transcription factor 3 (E2F3) mRNA, with YTHDC1 recognizing the m^6^A sites to facilitate its degradation, thereby blocking the G1/S phase transition of cell cycle and accelerating the senescent process of NP cells and progression of IDD (Liu et al., 2025c). In addition, Toll-like receptor 2 (TLR2) undergoes m^6^A modification, which is catalyzed by METTL3 and recognized by IGF2BP1 to enhance its stability, leading to upregulation of p53 and p21 expression and aggravating NP cell senescence and IDD (Ni et al., 2024). In summary, these *in vitro and in vivo* data highlight METTL3-mediated m^6^A modification as a driving force of the senescence of NP cells, providing an attractive target for therapeutic intervention of IDD.

### 5.4 METTL3 induces NP cell apoptosis

As a common mode of programmed cell death, apoptosis is characterized by chromosomal concentration, cell shrinkage, DNA degradation and apoptotic body formation, without eliciting inflammatory response. Nowadays, there has been a large body of evidence supporting the NP cell apoptosis as a critical contributor to IDD (Chen et al., 2022a; Jin et al., 2026; Zhang et al., 2026b). In human NP cells treated with TNF-α, METTL3 expression is significantly increased (Gao et al., 2022). Overexpression of METTL3 was found to promote the conversion of pri-miR-143-3p to miR-143-3p by recruiting DGCR8 in an m^6^A-dependent manner. Subsequently, miR-143-3p direct targets the 3′-UTR of SOX5, leading to induction of NP cell apoptosis and aggravation of IDD (Gao et al., 2022). Moreover, exogenous delivery of miR-143-3p inhibitor contributes to disc repair in a rat model of IDD (Gao et al., 2022). In another study, METTL3-mediated m^6^A epigenetic modification promotes miR-4534 maturation, which inactivates the sirtuin 6 (SIRT6)/nuclear factor erythroid 2-related factor 2 (Nrf2)/heme oxygenase-1 (HO-1) signaling cascade to exacerbate NP cell apoptosis and IDD (Ran et al., 2024). Collectively, these *in vitro and in vivo* observations suggest that blockade of the METTL3/miR-143-3p or METTL3/miR-4534 pathways may have a potential therapeutic benefit for IDD.

Alpha-ketoglutarate (α-KG), a precursor of glutamate and glutamine, functions as a significant intermediate product of the tricarboxylic acid cycle. As one of the best-investigated lncRNAs, metastasis-associated lung adenocarcinoma transcript 1 (MALAT1) is downregulated in NP cells isolated from IDD patients and plays a protective effect on IDD (Tao et al., 2024). More recently, Wu et al. reported that METTL3-mediated m^6^A methylation destabilizes MALAT1 to diminish its sponging of miR-23c, thereby resulting in isocitrate dehydrogenase 1 (IDH1) suppression (Wu et al., 2026). The decreased IDH1 levels inhibits α-KG production, facilitate reactive oxygen species (ROS) accumulation, triggers NP cell apoptosis, and ultimately aggravates disc disorganization in a mouse model of needle puncture-induced IDD (Wu et al., 2026). These authors also found that administration of α-KG alleviates IDD by suppressing ROS production and NP cell apoptosis (Wu et al., 2026). These results obtained from cultured cells and *in vivo* validation reveal a novel molecular cascade linking m^6^A to energy metabolism and IDD, and highlight the potential of targeting METTL3 or α-KG supplementation as therapeutic strategies. Although METTL3-mediated m^6^A methylation has a significant effect on MALAT1 stabilization, the precise mechanisms linking m^6^A deposition to MALAT1 decay are required to be elucidated in further research.

### 5.5 METTL3 facilitates mitochondrial dysfunction

Mitochondria act as a primary source of energy supply in cells and are essential for the production of a variety of biosynthetic intermediates. In addition to energy generation, mitochondria participate in a wide range of significant biological processes, such as cell differentiation, communication and death (Suomalainen and Nunnari, 2024; Al Amir Dache and Thierry, 2023). However, mitochondrial dysfunction is able to promote IDD development by affecting a number of pathophysiological processes. For example, mitochondrial impairment changes membrane permeability, which facilitates the release of large amounts of cytochrome C into the cytoplasm, activates the caspase cascade, and eventually causes disc cell apoptosis (Kong and Park, 2025). The mitochondrial homeostatic imbalance functions as a critical mediator of IVD cell senescence (Tang et al., 2026). Abnormal mitochondrial function can promote excessive ROS production, thereby leading to oxidative damage to disk cells (Zhang et al., 2026a). Mitophagy, a process that can selectively remove damaged or dysfunctional mitochondria via the autophagic machinery, plays a central role in maintaining mitochondrial quality control and homeostasis (Picca et al., 2023). Accumulating evidence has demonstrated that enhancing mitophagy significantly alleviates mitochondrial dysfunction and consequently inhibits ECM breakdown (Liang et al., 2026a; Zhang et al., 2022).

As an evolutionarily conserved mitochondrial enzyme, glutamate dehydrogenase 1 (GLUD1) can catalyze the conversion of L-glutamate to α-KG, thereby contributing to recycling amino acids into the tricarboxylic acid cycle so as to further produce energy (Bai et al., 2026). In human NP cells treated with hydrogen peroxide (H_2_O_2_), METTL3-mediated m^6^A methylation destabilize the GLUD1 mRNA by recruitment YTHDF2. Decreased GLUD1 expression reduces α-KG production and consequently impairs mitochondrial function (Chettiar and Dravid, 2026). This study establishes a mechanistic connection between METTL3 and IDD from the perspective of mitochondrial dysfunction, and provides a basis for METTL3-targeted intervention. Of note, METTL3-induced mitochondrial dysfunction is supported by cultured cells only. The *in vivo* validation is required to further determine mitochondrial dysfunction as an important mechanism linking METTL3 to IDD.

## 6 The diagnostic value and therapeutic potential of METTL3 in IDD

The diagnosis of IDD primarily depends on imaging techniques, particularly MRI. Currently, some biomarkers, such as serglycin, secreted frizzled-related protein 4 and IL-1 receptor 1, are being evaluated as novel methods for detecting IDD (Chen et al., 2024; Gao et al., 2022; Wu et al., 2025). Recently, Yang et al. reported that patients with severe IDD exhibit markedly elevated METTL3 levels compared with those with mild IDD, whereas there are no significant alterations in the expression levels of METTL4, WTAP, ALKBH5, FTO and YTHDF2 (Xu et al., 2025b). Another study showed that the expression levels of METTL3 gradually increase with aggravation of degeneration degree in human NP tissue (Yang et al., 2025b). These findings have established a positive correlation between METTL3 expression and degeneration severity. However, it should be emphasized that the current evidence is predominantly derived from cross-sectional observational studies, and no data are yet available regarding its diagnostic performance of METTL3 in clinical settings, such as sensitivity, specificity or optimal cut-off values. Also, there is a lack of assessment about its association with established imaging and clinical parameters. Therefore, whether METTL3 is appropriate for functioning as a diagnostic biomarker for IDD requires careful evaluation in the future studies.

As mentioned above, METTL3 is higher expressed in degenerative NP tissue and plays a causative role in the pathogenesis of IDD. Consequently, targeting METTL3 and its downstream pathways may represent a promising therapeutic approach for IDD. Currently, a variety of synthetic small-molecule inhibitors against METTL3 have emerged (Figure 6). For instance, STC-15 (also known as STM3480), which is a first-in-class, highly selective, and orally bioavailable METTL3 inhibitor, has recently been approved by FDA for phase 1b/2 clinical trial in patients with advanced malignancies (Yi et al., 2025). As a predecessor of STC-15, STM2457 can directly interact with the METTL3/METTL14 heterodimer for competition on m^6^A-containing substrate binding (Yankova et al., 2021). In H_2_O_2_-challenged human NP cells, prevention of METTL3 by STM2457 has been reported to effectively improve mitochondrial dysfunction, a critical event during IDD (Bai et al., 2026). This suggests that STM2457 may have potential therapeutic application in the management of IDD. STM3006 shares a largely conserved binding mode with STM2457, and is more potent than STM2457 in attenuating global RNA m^6^A levels. Like STM2457, UZH2 exhibits high inhibitory activity against METTL3 and high selectivity over other RNA methyltransferases, such as METTL16 and METTL1. Moreover, UZH2 can diminish the m^6^A/A level of polyadenylated RNA in dose-dependent manner and shows target engagement in MOLM-13 and PC-3 cell lines (Dolbois et al., 2021). UZH1a is also a SAM-competitive inhibitor and has a good cell permeability. Administration of UZH1a dramatically inhibits the growth of neuroblastoma in zebrafish, without mortality or hepatic toxicity (Lin et al., 2026). KH12 and elvitegravir are able to promote METTL3 degradation to attenuate its expression and global RNA methylation levels (Hwang et al., 2024; Liao et al., 2022), thereby providing a mechanism that distinct from small-molecule competitive inhibitors of METTL3. Additionally, eltrombopag is a selective allosteric inhibitor of METTL3 and has been shown to suppress the proliferation of acute myeloid leukemia cells through the hypomethylation of leukemogenic genes (Lee et al., 2022). It is worth noting that among these small-molecule METTL3 inhibitors, only STM2457 has been shown to inhibit IDD *in vitro*, and other inhibitors are derived from the non-IDD contexts, particularly malignant tumors. Given the fact that the biological behaviors of cancer cells are very different from IVD cells and their degenerative events, further studies are required to verify the efficacy and safety of these agents in animal models of IDD and early-phase clinical trials.

**FIGURE 6:**
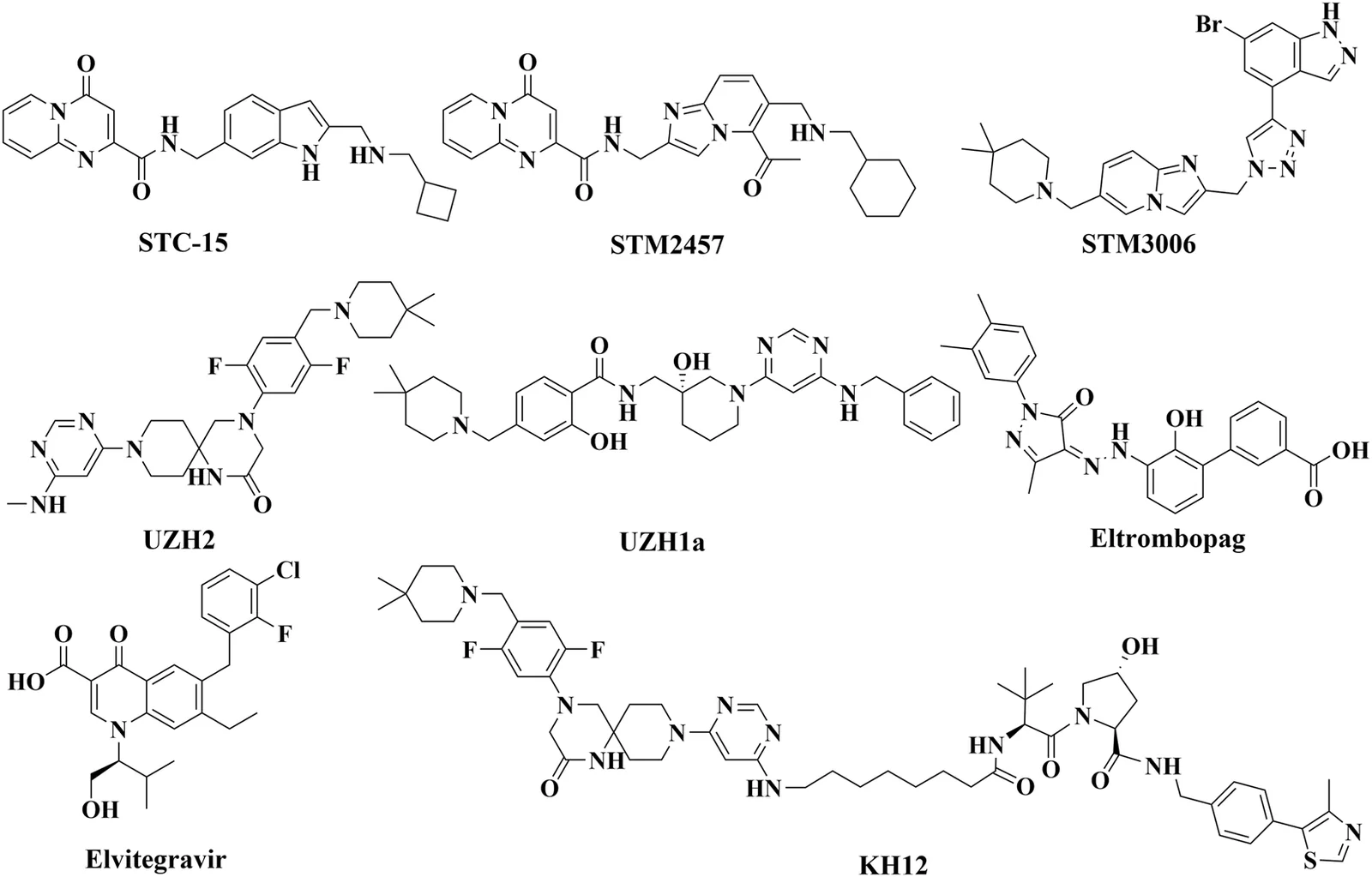
Chemical structures of eight small-molecule METTL3 inhibitors.

Despite targeting METTL3 holds significant promise for developing new therapies in IDD, several critical challenges should be addressed before clinical translation. First, considering that a variety of RNA molecules can be methylated by METTL3, the effectiveness, specificity and safety of METTL3-targeted epigenetic intervention for IDD should be carefully evaluated. Second, it is essential to develop the delivery technologies with high tissue specificity because METTL3 is ubiquitously expressed in tissues. Systemic administration may exert a significant impact on the biological behavior of normal tissues, leading to off-target effects. Thus, how METTL3-targeted drugs are precisely delivered to degenerative IVD tissue for enhancing their efficacy needs to be resolved. Third, given that there is a significant cross-talk between m^6^A and other RNA methylation modifications such as 5-methylcytosine and N1-methyladenosine (Song et al., 2022; Wang et al., 2022b), blockade of METTL3-mediated m^6^A methylation may affect other RNA modifications and consequently results in unexpected effects. This needs to be elucidated in future research. Finally, it is also important to explore the feasibility of METTL3-targeted drugs in combination with established therapies for IDD.

## 7 Conclusion and future directions

METTL3, the sole catalytic component of the methyltransferase complex, has a SAM-binding site and can transfers a methyl group from SAM to N^6^-adenosine for installing m^6^A. METTL3 is ubiquitously expressed, and it is modulated by a variety of factors at the transcriptional and post-transcriptional levels. Aberrant expression of METTL3 is frequently observed in degenerative IVD tissue and exhibits a correlation with degeneration degree and age. Importantly, METTL3-mediated m^6^A modification has been shown to promote the onset and development of IDD through multiple approaches. This not only broaden our understanding about RNA epigenetic regulation in the pathogenesis of IDD, but also offers a potential biomarker and therapeutic target for IDD.

At present, m^6^A modification has become a research hotspot. Despite great progress has been made in this field over the past decade, the current research on the role of METTL3 in IDD is still in the preliminary stage. There are a lot of issues that need to be addressed. Up to now, the majority of studies primarily investigate the mechanisms of action of METTL3. Nevertheless, only few studies focus on the application of targeting METTL3, particularly the use of small-molecule inhibitors in IDD. Thus, future research should more explore the efficacy and safety of small-molecule METTL3 inhibitors in the management of IDD, which is essential for clinical translation of METTL3-targeted epigenetic intervention. Given that m^6^A modification is a complex process involving multiple modulators, it is important to reveal the competitive or synergistic effects among these regulatory factors during IDD. Studies from our group and other teams have demonstrated that like apoptosis, pyroptosis and ferroptosis in IVD cells also contribute to the development of IDD (Wu et al., 2023; Yang et al., 2022b; Zhan et al., 2024; Sun et al., 2025). It has been reported that METTL14 is significantly upregulated in NP cells isolated from IVDD patients, and METTL14-induced m^6^A modification enhances the stability of NLRP3 mRNA in an IGFBP2-dependent manner to trigger pyroptotic NP cell death (Yuan et al., 2021). Moreover, there is accumulating evidence that METTL3 serves as an important regulator of pyroptosis and ferroptosis in multiple cell types, such as glioblastoma cells and podocytes (Xu et al., 2025a; Meng et al., 2024). Thus, it is possible that METTL3 is potentially implicated in the pyroptosis and ferroptosis of IVD cells. Future research in this field is of great importance to deepen our understanding of the role of METTL3 in IDD. METTL3 is able to interact with eukaryotic translation initiation factor 3 subunit h so as to enhance ribosome recycling and translation efficiency, which is independent of its catalytic activity in the context of cancers (Lin et al., 2016; Choe et al., 2018). The m^6^A-independent functions of METTL3 may also be involved in the pathogenesis of IDD, which needs to be investigated. It is well known that both AF and CEPs can suffer from degeneration besides NP tissue. Of note, current evidence is predominantly derived from NP models and there is considerably less information on AF and CEP cells. It is important to uncover the influence of METTL3 on AF and CEP degeneration. In summary, the investigation about METTL3-mediated m^6^A methylation in the pathogenesis of IDD is currently undergoing rapid development, although the specific role and precise mechanisms require further elucidation. With continued efforts, METTL3 may become a novel and promising target for the prevention and treatment of IDD.
